# Viral and Immunologic Factors Associated with Fatal Outcome of Patients with Severe Fever with Thrombocytopenia Syndrome in Korea

**DOI:** 10.3390/v13122351

**Published:** 2021-11-23

**Authors:** Ji-Soo Kwon, Sol Jin, Ji-Yeun Kim, Sang-Hyun Ra, Taeeun Kim, Se-Yoon Park, Min-Chul Kim, Seong-Yeon Park, Dasarang Kim, Hye-Hee Cha, Hyun-Jung Lee, Min-Jae Kim, Yong-Pil Chong, Sang-Oh Lee, Sang-Ho Choi, Yang-Soo Kim, Keun-Hwa Lee, Sun-Ho Kee, Sung-Han Kim

**Affiliations:** 1 Department of Infectious Diseases, Asan Medical Center, University of Ulsan College of Medicine, Seoul 05505, Korea; kwonjs92@kaist.ac.kr (J.-S.K.); solb613@hanmail.net (S.J.); aeki22@snu.ac.kr (J.-Y.K.); jesus4274@naver.com (S.-H.R.); heyhe0102@naver.com (H.-H.C.); silverspec@naver.com (H.-J.L.); nahani99@gmail.com (M.-J.K.); drchong@amc.seoul.kr (Y.-P.C.); soleemd@amc.seoul.kr (S.-O.L.); sangho@amc.seoul.kr (S.-H.C.); yskim@amc.seoul.kr (Y.-S.K.); 2 Division of Infectious Diseases, Gyeongsang National University Hospital, Gyeongsang National University School of Medicine, Jinju 52727, Korea; sleepju@naver.com; 3 Division of Infectious Diseases, Department of Internal Medicine, Soonchunhyang University Seoul Hospital, Soonchunhyang University College of Medicine, Seoul 04401, Korea; livinwill2@gmail.com; 4 Division of Infectious Diseases, Chung-Ang University Hospital, Seoul 06973, Korea; pour-soi@hanmail.net; 5 Department of Infectious Diseases, Dongguk University Ilsan Hospital, Goyang 10326, Korea; psy99ch@hanmail.net; 6 Department of Microbiology, Institute for Viral Diseases, College of Medicine, Korea University, Seoul 02841, Korea; ddeang233@naver.com (D.K.); keesh@korea.ac.kr (S.-H.K.); 7 Department of Microbiology, College of Medicine, Hanyang University, Seoul 04763, Korea; yomust7@gmail.com

**Keywords:** SFTS phlebovirus, fatal outcome, cytokines, chemokines, humoral immunity

## Abstract

Significant progress has been made on the molecular biology of the severe fever with thrombopenia virus (SFTSV); however, many parts of the pathophysiological mechanisms of mortality in SFTS remain unclear. In this study, we investigated virologic and immunologic factors for fatal outcomes of patients with SFTS. We prospectively enrolled SFTS patients admitted from July 2015 to October 2020. Plasma samples were subjected to SFTSV RNA RT-PCR, multiplex microbead immunoassay for 17 cytokines, and IFA assay. A total of 44 SFTS patients were enrolled, including 37 (84.1%) survivors and 7 (15.9%) non-survivors. Non-survivors had a 2.5 times higher plasma SFTSV load than survivors at admission (*p* < 0.001), and the viral load in non-survivors increased progressively during hospitalization. In addition, non-survivors did not develop adequate anti-SFTSV IgG, whereas survivors exhibited anti-SFTSV IgG during hospitalization. IFN-α, IL-10, IP-10, IFN-γ, IL-6, IL-8, MCP-1, MIP-1α, and G-CSF were significantly elevated in non-survivors compared to survivors and did not revert to normal ranges during hospitalization (*p* < 0.05). Severe signs of inflammation such as a high plasma concentration of IFN-α, IL-10, IP-10, IFN-γ, IL-6, IL-8, MCP-1, MIP-1α, and G-CSF, poor viral control, and inadequate antibody response during the disease course were associated with mortality in SFTS patients.

## 1. Introduction

Severe fever with thrombocytopenia syndrome (SFTS) is an emerging tick-borne disease in East Asia, primarily in China, Korea, and Japan. SFTS virus (SFTSV), now renamed Dabie bandavirus, has been identified as the causative agent. This virus is a novel phlebovirus of the family Phenuiviridae, order Bunyavirales, and is usually transmitted by a tick such as *Haemaphysalis longicornis* [1]. The disease is characterized by thrombocytopenia and leukopenia following the onset of fever, as well as respiratory or gastrointestinal symptoms, and multiple organ failure can develop in severe cases. Since its first description, the incidence of SFTS has increased in China, Japan, and South Korea, and the case fatality ratio has been reported to be 16.2–32.6% [2,3]. There has been significant progress in our understanding of the molecular biology of SFTSV. Recent research findings have indicated that SFTSV can enter various cell types via pH-dependent endocytosis and interfere with the signaling pathways of the innate and adaptive immunity [4,5,6,7,8,9]. However, many aspects of the pathophysiological mechanisms that lead to fatal outcomes in SFTS patients have remained unclear.

A cytokine storm was reported to be one of the major pathophysiological features underlying the high mortality rate for SFTS [10,11]. This dysregulation of the immune system via hypercytokinemia can cause leukopenia and thrombocytopenia due to peripheral destruction of platelets and/or bone marrow suppression. Recent studies have reported that some cytokines are elevated in severe cases and in the acute phase of this disease [10,12,13,14,15]. Our previous study also found an initial hypercytokinemia in a small number of SFTS patients (*n* = 11) [16]. However, limited data are available on the detailed kinetics of the cytokine profiles in SFTS patients with fatal outcomes, compared to those who survive this infection [10,12,13]. In the present study, we analyzed the cytokine profiles, viral load, and antibody responses to SFTSV during the disease course and identified some of the factors that can lead to fatal outcomes of patients with SFTS.

## 2. Materials and Methods

### 2.1. Patients and Clinical Samples

We prospectively enrolled 44 confirmed cases of SFTS admitted to 5 university-affiliated hospitals in South Korea from June 2015 to October 2020: Asan Medical Center, Gyeongsang National University Hospital, Soonchunhyang University Seoul Hospital, Chung-Ang University Hospital, and Dongguk University Ilsan Hospital. SFTSV infection was confirmed by detection of viral RNA with real-time reverse transcription polymerase chain reaction (RT-PCR). During the hospitalization period, plasma samples were obtained from each patient and collected in an EDTA-treated vacutainer. The plasma was immediately separated from the whole blood and frozen at −80 °C until further analysis. The study protocol was approved by the respective institutional review boards of participating hospitals (Asan Medical Center IRB no. 2016-0748 (15 July 2016); Gyeongsang National University Hospital IRB no. 2019-10-019 (28 November 2019); Soonchunhyang University Seoul Hospital IRB no. 2016-09-001 (30 November 2016); Chung-Ang University Hospital IRB no. 1970-002-376 (18 November 2019); Dongguk University Ilsan Hospital IRB no. 2016-01-088 (25 August 2016)).

### 2.2. Quantification of Viral RNA

The viral load of patients with SFTS was measured by using one-step multiplex real-time RT-PCR. Viral RNA was extracted from plasma samples using the Qiagen RNeasy Mini Kit (Qiagen, Hilden, Germany). Segment S and M genes were detected to quantify the viral load, and the human β-actin gene was used as an internal control. The sequences of the primers and probes used in this study were as follows: Seg S Forward 5′-CGAGAGAGCTGGCCTATGAA-3′, Reverse 5′-TTCCCTGATGCCTTGACGAT-3′, and Probe 5′-FAM-TGTCTTTGCCCTGACTCGAGGCA-BHQ1-3′; Seg M Forward 5′-ATGCTTGTCGTGAAGAAGGC-3′, Reverse 5′-CTAGACTTCCCACTGCCACA-3′, and Probe 5′-Cy5-ACTTTTGATGGATACGTAGGCTGGGGC-BHQ2-3′; β-actin Forward 5′-ACTAACACTGGCTCGTGTGA-3′, Reverse 5′-CTTGGGATGGGGAGTCTGTT-3′, and Probe 5′-HEX-AGGCTGGTGTAAAGCGGCCTTGG-BHQ1-3′. The reaction mixture was prepared with LightCycler Multiplex RNA Virus Master (Roche Diagnostics, Indianapolis, IN, USA), and RT-PCR was conducted using a LightCycler 96 system (Roche Diagnostics) in accordance with the manufacturer’s instructions. The SFTSV RNA copy number was determined on the basis of a standard curve generated from the Ct values of in vitro transcript RNA. The detection limit of the RT-PCR was 4.3 copies/μL of plasma sample. The method is described in detail in our previous study [17].

### 2.3. Measurement of Plasma Cytokines

We simultaneously measured the levels of 17 selected cytokines in the plasma samples of the SFTS subjects using a cytometric bead array (BD biosciences, San Jose, CA, USA), as described in our previous study [16]. In brief, capture beads for the following cytokines were incubated with the plasma samples: granulocyte colony-stimulating factor (G-CSF), granulocyte macrophage colony stimulating factor (GM-CSF), interferon (IFN)-α, IFN-γ, tumor necrosis factor (TNF)-α, interleukin (IL)-1β, IL-6, IL-8, IL-10, IL-12p40, IL-13, IL-17A, monocyte chemotactic protein (MCP)-1, macrophage inflammatory protein (MIP)-1α, regulated on activation and normally T cell expressed and secreted (RANTES), IFNγ-induced protein (IP)-10, and vascular endothelial growth factor (VEGF). PE-conjugated antibodies were used to detect the proteins on the capture beads following washing out of the reagents and unbound antibodies. Data were acquired using a FACS CANTO II flow cytometer, FACS Diva software (ver 8.0, BD Biosciences, San Jose, CA, USA), and FlowJo software (ver 10.7.1, FlowJo LLC, Ashland, OR, USA).

### 2.4. Measurement of Anti-SFTSV IgG

Serological testing for the presence of anti-SFTSV IgG was performed using an immunofluorescence antibody (IFA) assay as previously described [18]. For IFA, Vero E6 cells infected with SFTSV were grown in a 5% CO_2_ incubator at 37 °C for 5 days. For preparation of IFA antigens, cells were harvested, coated onto Teflon-coated well slides, and then fixed with acetone. IFA assay was carried out using the patient’s serum as the primary antibody and fluorescein-labeled antihuman IgG secondary antibodies (Thermo Fisher Scientific, Waltham, MA, USA). A monoclonal anti-SFTSV N antibody, manufactured in our laboratory, was used as the positive control.

### 2.5. Statistical Analysis

For statistical analyses, categorical variables were compared using Fisher’s exact test or the χ^2^ test, and continuous variables were compared with the Mann–Whitney U test. The Spearman test was used to calculate the correlation coefficient between cytokine/chemokine levels and viral RNA load. Principal component analysis (PCA) was used to assess the relationship between multiple cytokines/chemokines and the outcome of SFTS. All tests of significance were two-tailed, and p values less than 0.05 were considered statistically significant. Statistical analyses were performed using GraphPad Prism 9.1.2 (GraphPad Software, Inc., La Jolla, CA, USA).

## 3. Results

### 3.1. Clinical Characteristics of the Patients

Forty-four patients with SFTS confirmed by an SFTSV-specific RT-PCR result from plasma specimens were enrolled, including 37 (84.1%) survivors and 7 (15.9%) non-survivors. Among these patients, 27 (61.4%) were men, and the mean age (±standard deviation) was 63.8 (±10.5) years. Twenty-five (56.8%) patients had no underlying diseases, but the others had a broad range of underlying diseases including diabetes mellitus (*n* = 12, 27.3%), a solid tumor (*n* = 3, 6.8%), chronic liver disease (*n* = 3, 6.8%), chronic lung disease (*n* = 4, 9.1%), and autoimmune disease (*n* = 1, 2.3%). Detailed baseline characteristics of these patients are provided in Table 1.

### 3.2. Viral and Immunological Factors Associated with Mortality

**Figure 1:**
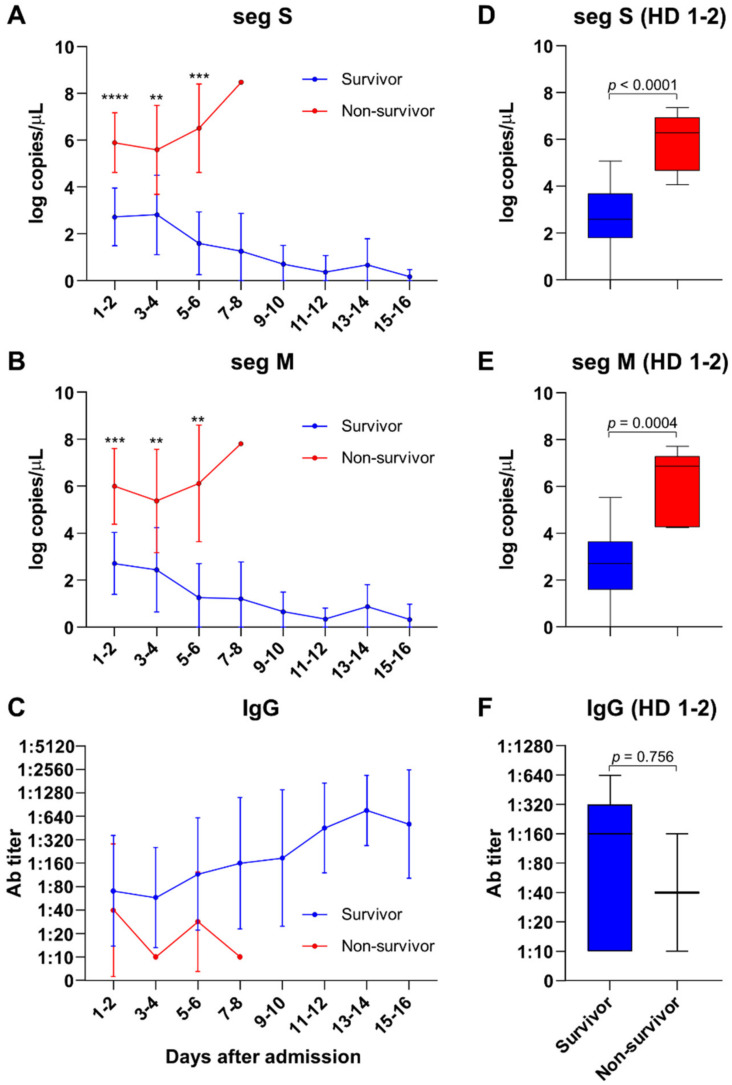
Plasma SFTS viral load and antibody titer in patients with SFTS. Both segment S (seg S) and M (seg M) titers were significantly higher in non-survivors than in survivors and increased during hospitalization (**A**,**B**). The SFTS viral load (hospital days 1–2) was significantly higher in non-survivors (**D**,**E**). The plasma antibody titer against SFTSV increased in survivors during hospitalization but decreased in non-survivors (**C**). There were no differences between the initial (hospital days 1–2) anti-SFTSV-IgG titers of the survivors and non-survivors (**F**). Blue, survivor; red, non-survivor. ** *p* < 0.01, *** *p* < 0.001, **** *p* < 0.0001.

The detailed kinetics of viral load and antibody response in the SFTS patients after admission are shown in Figure 1. The non-survivors had median viral loads that were about 2.5 times higher than those of the survivors, and these loads continuously increased during the hospitalization period (Figure 1A,B). The peak viral load in the survivors was observed at hospital days 1–2, and the amount of viral RNA decreased over time in these cases. The initial viral loads were also significantly higher in patients who died compared with those who recovered (*p* < 0.0001 for seg S, *p* = 0.0004 for seg M, Figure 1D,E). The median initial viral load in the survivors was 2.59 log copies/µL (IQR, 1.78–3.68) for segment S, and 2.70 log copies/µL (IQR, 1.59–3.64) for segment M. In the patients who died, however, the initial titer for segment S was 6.28 log copies/µL (IQR, 4.67–6.94), and that for segment M was 6.86 log copies/µL (IQR, 7.29). An anti-SFTSV IgG response developed in survivors but was found to be impaired in the non-survivors (Figure 1C,F).

**Figure 2:**
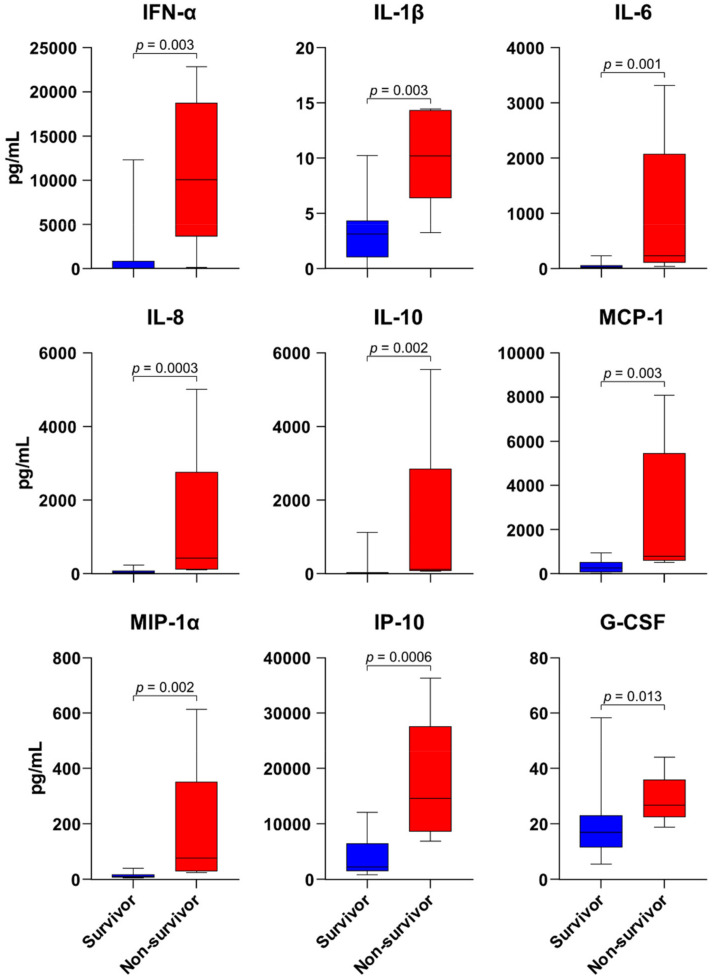
Initial plasma concentrations of cytokines and chemokines in patients with SFTS. The plasma concentrations of IFN-α, IL-1β, IL-6, IL-8, IL-10, MCP-1, MIP-1α, IP-10, and G-CSF on hospital days 1–2 in non-survivors were significantly higher than those in the survivors. Blue, survivor; red, non-survivor.

**Figure 3:**
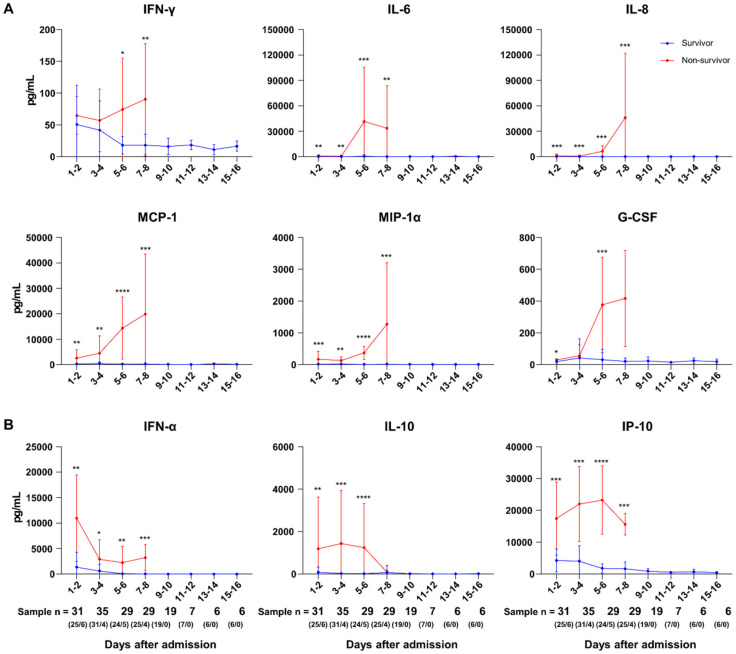
Plasma concentrations of cytokines and chemokines in patients with SFTS. IFN-γ, IL-6, IL-8, MCP-1, MIP-1α, and G-CSF levels gradually increased during hospitalization (**A**), whereas IFN-α, IL-10, and IP-10 levels decreased or did not change (**B**). The numbers of analyzed samples were as follows: HD 1–2, *n* = 31 (survivor/non-survivor, 25/6); HD 3–4, *n* = 35 (31/4); HD 5–6, *n* = 29 (24/5); HD 7–8, *n* = 29 (25/4); HD 9–10, *n* = 19 (19/0); HD 11–12, *n* = 7 (7/0); HD 13–14, *n* = 6 (6/0); HD 15–16, *n* = 6 (6/0). * *p* < 0.05, ** *p* < 0.01, *** *p* < 0.001, **** *p* < 0.0001.

A total of 162 plasma specimens from the SFTS subjects were available for multiplex cytokine bead array analysis. Among the 17 cytokines measured in these assays, the plasma concentrations of IFN-α, IL-1β, IL-6, IL-8, IL-10, MCP-1, MIP-1α, IP-10, and G-CSF were found to be significantly higher in the non-survivors at admission (Figure 2). In addition, the plasma concentrations of these cytokines were markedly higher in non-survivors throughout the disease course. During the disease course, IFN-γ, IL-6, IL-8, MCP-1, MIP-1α, and G-CSF were elevated and reached a maximal level in non-survivors after day 5 of hospitalization (Figure 3A). On the other hand, IFN-α was at its highest level on the day of admission and decreased throughout the disease in all patients. The plasma concentrations of IL-10 and IP-10 did not show significant changes during hospitalization (Figure 3B). The levels of these cytokines did not return to within a normal range in either survivors or non-survivors. The RANTES level in non-survivors decreased below its normal range during the disease course and was significantly lower in non-survivors compared with survivors, at hospital days 7–8 (*p* = 0.001). As shown in Supplementary Table S1, IFN-α, IFN-γ, IL-10, MCP-1, IL-8, IP-10, IL-6, MIP-1α, and G-CSF were significantly correlated with the initial viral load, while RANTES and VEGF showed inverse correlations with the initial viral load. In addition, when the cytokines were compared on the basis of intensive care unit (ICU) admission, the plasma concentrations of IL-6, IL-8, IL-10, and MIP-1a were significantly higher in ICU-admitted patients than in others (Supplementary Table S2). Moreover, the plasma concentrations of IL-6 and IL-8 were significantly higher in patients with CNS involvement than in others (Supplementary Table S3). Therefore, proinflammatory cytokines IL-6 and IL-8 may contribute to severity in survivors.

In addition, principal component analysis (PCA) was performed to identify the prognostic marker for SFTS (Supplementary Figure S1). The data of non-survivors and survivors had different distributions on the plot. Most cytokines and chemokines had a positive relation with the first principal component (PC1); therefore, PC1 was associated with SFTSV infection. The second principal component (PC2) had a positive relation with the data of non-survivors, and thus PC2 was associated with the fatality of SFTS. The cytokines most related to PC1 were identified in the order of MCP-1, MIP-1α, IL-1β, and IL-8. In addition, IP-10, MCP-1, IL-10, IFN-α, and IFN-γ were identified to be related to PC2 in that order. Therefore, these cytokines might be useful as prognostic markers for SFTS.

## 4. Discussion

It is well known that an excessive release of cytokines and chemokines by activated immune cells and infected cells is involved in immunopathology and the development of organ dysfunction. Our findings in this study, namely, that there are high plasma concentrations of IFN-α, IL-10, IP-10, IFN-γ, IL-6, IL-8, MCP-1, MIP-1α, G-CSF, and viral RNA in non-surviving cases of SFTS, suggest that excessive cytokine release and uncontrolled viremia were critical determinants of these fatal outcomes.

IL-10 plays immunoregulatory roles during a variety of infections [19]. IL-10 is expressed by almost all subsets of leukocytes and protects the host from tissue damage resulting from excessive proinflammatory responses during infection. In certain types of infection, however, the cytokine may interfere with pathogen clearance and contribute to pathogenesis. Polymorphisms in the IL-10 gene or increased IL-10 production can increase host susceptibility to a wide variety of infections in humans and in animal models [20]. In addition, the cytokine is critical to the establishment of T cell exhaustion and viral persistence [21]. In terms of SFTSV infection, our present results are comparable with those of several studies that have consistently reported that IL-10 is higher in severe and fatal cases than in mild cases [10,12,13,14]. Choi et al. suggested that a local immunosuppressive environment created by the upregulation of IL-10 following TPL2 activation by the non-structural (NS) protein of SFTSV enables vigorous viral replication, leading to viral pathogenesis [22]. Although IL-10 is commonly known to promote B cell survival and plasma cell differentiation, its upregulation does not seem to affect the increase in humoral immune response in non-surviving SFTS patients. In a previous study, Song et al. suggested that IL-10 contributes to disrupted B cell immunity by suppressing germinal center formation and inhibiting the differentiation of dendritic cells [8]. It is worth noting that there was a decrease in IL-10 in non-surviving SFTS patients (Figure 1 and Figure 3), despite a high viral load. The initial increase in immunosuppressive IL-10 production induced by an SFTS non-structural protein may be considered as a key immune evasive mechanism of SFTS [23]. It is thus unclear why IL-10 decreases in the late course of fatal SFTS. However, a limited number of patients were included in this non-survivor late course (HD# 7-8), and thus further studies are needed on whether there is a decrease in IL-10 in the late course of fatal SFTS. Taken together, the evidence to date suggests that elevated IL-10 may play an important role in the pathophysiology leading to fatal outcomes in patients with SFTS.

IFN-α, a member of the type I IFNs, is a cytokine produced by virus-infected cells and activated immune cells. Exposure of cells to IFNs induces an antiviral state and prevention of productive viral infection. Type I IFN is thought to play a protective role during phlebovirus infection, and these viruses have counterstrategies to delay or prevent type I IFN responses. It has been reported that the NS proteins of SFTSV and other phleboviruses disrupt type I IFN by various mechanisms [24]. Chaudhary et al. and Ning et al. observed that the SFTSV NS protein suppresses type I IFN signaling by reducing STAT1 and STAT2 activity, leading to an inhibition of downstream interferon-stimulated gene responses [5,6,7,25]. Hence, an initial elevation in IFN-α in patients with a high SFTSV load would seem to be counterintuitive. However, an early appropriate type I IFN response may be important to control viral replication, but delayed type I IFN with an excessive response due to extensive tissue damage may be detrimental for tissue inflammation, as shown in our non-surviving patients who initially presented as having multi-organ failure. Further research on the role of type I IFN in SFTSV infection is needed [15,16].

Significant roles of MIP-1α, MCP-1, and IP-10 and their receptors in viral infection have been reported [26]. Elevated chemokine levels in fatal and severe SFTS were also described previously [10,12,27]. In addition, we found in our present analysis that increased chemokine levels returned to a normal range in patients who survived SFTS, but not in non-survivors. The consistent finding of high chemokine levels in fatal and severe SFTS cases may indicate the importance of the innate immune response in this disease.

There were several noteworthy limitations to the present study. First, we analyzed a relatively small number of patients (*n* = 44) and thus could not perform multivariate analysis to identify any confounding factors. Second, we did not examine the adaptive cellular immune response that may well contribute to a resolution of this viral infection. More in-depth investigations on cellular and humoral immune responses against SFTSV are needed, as they may assist with the development of vaccines and therapeutic agents. Finally, our findings suggest that uncontrolled viremia or high cytokine/chemokine releases may be associated with pathophysiologic mechanisms resulting in a fatal outcome. However, the pathophysiologic mechanism of SFTS is largely unknown. The patients with fatal SFTS merely had a more severe disease course with more upregulated immunologic parameters. Thus, uncontrolled viremia or high cytokine/chemokine releases might be intermediate variables in the causal pathway, as with septic shock in bacteremia patients [28]. Further studies are needed in this area.

## 5. Conclusions

In conclusion, the current study shows that severe inflammation reflected in a high plasma concentration of cytokines, poorly controlled viral replication, and an impaired humoral response is associated with fatal outcomes in SFTS patients.

## Figures and Tables

**Table 1:** Baseline characteristics of the study patients with SFTS.

	Total (*n* = 44)	Survivor (*n* = 37)	Non-Survivor (*n* = 7)	*p* Value
Age (years), mean ± SD	63.8 ± 10.5	62.4 ± 10.0	71.0 ± 10.9	0.031
Male gender	27 (61.4)	21 (56.8)	6 (85.7)	0.220
Season (months)				
Spring–Summer (3–8)	21 (47.7)	15 (40.5)	6 (85.7)	0.042
Fall (9–11)	23 (52.3)	22 (59.5)	1 (14.3)	
Eschar	11 (25.0)	9 (24.3)	2 (28.6)	1.000
Clinical characteristics				
Fever	43 (97.7)	36 (97.3)	7 (100.0)	1.000
Skin rash	6 (13.6)	5 (13.5)	1 (14.3)	1.000
Bleeding	6 (13.6)	5 (13.5)	1 (14.3)	1.000
Myalgia	25 (56.8)	22 (59.5)	3 (42.9)	0.443
Anorexia/General weakness	34 (77.3)	29 (78.4)	5 (71.4)	0.649
Nausea/Vomiting	21 (47.7)	18 (48.6)	3 (42.9)	1.000
Diarrhea	19 (43.2)	16 (43.2)	3 (42.9)	1.000
Stomachache	13 (29.5)	10 (27.0)	3 (42.9)	0.404
Dyspnea	13 (29.5)	10 (27.0)	3 (42.9)	0.404
Altered mental status	24 (54.5)	17 (45.9)	7 (100.0)	0.011
Concomitant infection	12 (27.3)	9 (24.3)	3 (42.9)	0.369
Underlying diseases				
Previously healthy	25 (56.8)	19 (51.4)	6 (85.7)	0.119
Diabetes mellitus	12 (27.3)	9 (24.3)	3 (42.9)	0.369
Solid tumor	3 (6.8)	2 (5.4)	1 (14.3)	0.413
Chronic liver disease	3 (6.8)	1 (2.7)	2 (28.6)	0.061
Chronic kidney disease	0	0	0	
Chronic lung disease	4 (9.1)	2 (5.4)	2 (28.6)	0.113
Autoimmune disease	1 (2.3)	0	1 (14.3)	0.159
Immunosuppressive condition	0	0	0	
Laboratory findings				
WBC (/μL), median (IQR)	1800 (1070–2823)	1800 (1080–3065)	1800 (1000–2500)	0.712
Hemoglobin (g/dL), median (IQR)	14.0 (12.9–15.1)	14.0 (12.2–15.1)	15.0 (17.7–15.2)	0.051
Platelets × 10^3^(/μL), median (IQR)	57.5 (42.0–77.5)	59.0 (42.0–80.0)	53.0 (33.0–66.0)	0.577
BUN (mg/dL), median (IQR)	17.0 (12.0–23.8)	14.0 (10.5–21.5)	26.0 (20.0–28.0)	0.013
Creatinine (mg/dL), median (IQR)	0.84 (0.66–1.13)	0.80 (0.64–1.08)	1.33 (0.89–1.72)	0.017
AST (IU/L), median (IQR)	214.0 (121.8–429.3)	213.0 (127.5–355.5)	484.0 (51.0–2005.0)	0.466
ALT (IU/L), median (IQR)	103.0 (69.0–184.5)	101.0 (59.5–138.5)	197.0 (69.0–535.0)	0.286
CRP (mg/dL), median (IQR)	0.46 (0.10–0.98)	0.30 (0.10–0.80)	1.30 (0.42–5.70)	0.070
Time from symptom onset to admission (days), median (IQR)	6.0 (4.3–7.0)	7.0 (4.0–7.0)	5.0 (5.0–6.0)	0.476
Time from hospital admission to defervescence (days), median (IQR) ^†^	3.0 (2.0–5.8)	3.5 (1.8–6.3)	3.0 (1.8–5.0)	0.715
Time from hospital admission to hospital discharge or death (days), median (IQR)	10.0 (7.3–14.5)	11.0 (9.0–15.0)	5.0 (3.0–7.0)	<0.0001
Clinical course				
ICU admission	17 (38.6)	10 (27.0)	7 (100.0)	0.0005
Treatment				
Doxycycline	37 (84.1)	32 (86.5)	5 (71.4)	0.307
Ribavirin	18 (40.9)	15 (40.5)	3 (42.9)	1.000
Plasma exchange	29 (65.9)	23 (62.2)	6 (85.7)	0.393
Convalescent plasma therapy	3 (6.8)	2 (5.4)	1 (14.3)	0.413

^†^ Data missing for 5 survivors and 1 non-survivor. Abbreviations: WBC, white blood cells; BUN, blood urea nitrogen; AST, aspartate transaminase; ALT, alanine transaminase; CRP, C-reactive protein; ICU, intensive care unit; SD, standard deviation; IQR, interquartile range.

## Data Availability

Data sharing is not applicable.

## Supplementary Materials

The following are available at https://www.mdpi.com/article/10.3390/v13122351/s1, Figure S1: Principal component analysis (PCA) biplot of the entire dataset from 44 SFTS patients, plotted along PC1 and PC2. Table S1: Correlation of cytokine and chemokine levels with levels of initial viral RNA from blood in patients with SFTS., Table S2: Comparison of initial cytokine and chemokine levels of survivor SFTS patients upon ICU admission., Table S3: Comparison of initial cytokine and chemokine levels of survivor SFTS patients with CNS involvement.
