# Supplementary material for: Viral and Immunologic Factors Associated with Fatal Outcome of Patients with Severe Fever with Thrombocytopenia Syndrome in Korea
Source: Viruses. 2021 Nov 23;13(12):2351. doi: 10.3390/v13122351 (PMC8703577; doi:10.3390/v13122351)
Supplement: Supplementary file 1 [file viruses-13-02351-s001.zip › viruses-1449524-supplementary.pdf]

Table S1. Correlation of cytokine and chemokine levels with levels of initial viral RNA from blood in patients with SFTS.

Cytokines/Chemokines	Spearman r (95% CI)	p Value
GM-CSF	0.163 (−0.025–0.339)	0.079
IFN-γ	0.546 (0.400–0.665)	<0.0001
IL-6	0.432 (0.267–0.573)	<0.0001
IL-8	0.515 (0.363–0.640)	<0.0001
IP-10	0.797 (0.717–0.857)	<0.0001
MCP-1	0.688 (0.576–0.776)	<0.0001
RANTES	−0.321 (−0.479–0.143)	0.0004
VEGF	−0.199 (−0.372–0.013)	0.032
G-CSF	0.317 (0.138–0.475)	0.0005
IFN-α	0.730 (0.629–0.807)	<0.0001
IL-10	0.649 (0.526–0.746)	<0.0001
IL-12p40	0.059 (−0.129–0.243)	0.528
IL-13	−0.004 (−0.191–0.182)	0.961
IL-17A	0.048 (−0.141–0.232)	0.611
IL-1β	0.107 (−0.081–0.288)	0.250
MIP-1α	0.596 (0.461–0.705)	<0.0001
TNFα	0.074 (−0.114–0.257)	0.427

Table S2. Comparison of initial cytokine and chemokine levels of survivor SFTS patients upon ICU admission.

	ICU– (n = 27)	ICU+ (n = 10)	p Value
Days from admission (days), median (IQR)	2.0 (1.0–3.0)	2.0 (1.75–5.75)	0.498
GM-CSF (pg/mL), median (IQR)	0.0 (0.0–4.2)	0.0 (0.0–6.7)	0.540
IFN-γ (pg/mL), median (IQR)	26.0 (12.2–53.8)	33.9 (19.7–158.5)	0.268
IL-6 (pg/mL), median (IQR)	13.7 (7.5–33.0)	112.8 (87.3–147.2)	<0.0001
IL-8 (pg/mL), median (IQR)	28.6 (16.5–43.2)	91.2 (41.7–241.0)	0.002
IP-10 (pg/mL), median (IQR)	2152 (1527–4438)	8269 (1303–11429)	0.091
MCP-1 (pg/mL), median (IQR)	181.8 (62.6–436.7)	423.0 (110.2–827.0)	0.137
RANTES (pg/mL), median (IQR)	5904 (2952–11893)	2365 (1720–4042)	0.052
VEGF (pg/mL), median (IQR)	20.3 (11.6–65.7)	18.3 (0–32.0)	0.394
G-CSF (pg/mL), median (IQR)	17.0 (10.8–23.0)	15.0 (12.7–36.8)	0.516
IFN-α (pg/mL), median (IQR)	23.0 (10.6–473.8)	338.7 (81.4–5121.0)	0.051
IL-10 (pg/mL), median (IQR)	14.3 (6.2–27.7)	51.9 (10.1–98.8)	0.047
IL-12p40 (pg/mL), median (IQR)	119.5 (83.9–216.7)	112.8 (61.4–220.5)	0.668
IL-13 (pg/mL), median (IQR)	2.4 (1.4–4.0)	1.6 (0.3–4.4)	0.384
IL-17A (pg/mL), median (IQR)	4.4 (2.3–10.2)	9.3 (1.5–14.5)	0.644
IL-1β (pg/mL), median (IQR)	3.6 (2.0–7.5)	3.1 (2.2–6.9)	0.980
MIP-1α (pg/mL), median (IQR)	9.8 (7.3–15.0)	18.6 (11.7–44.5)	0.008
TNFα (pg/mL), median (IQR)	2.5 (0.0–12.8)	5.1 (0.0–8.9)	0.645

Table S3. Comparison of initial cytokine and chemokine levels of survivor SFTS patients with CNS involvement.

	CNS– (*n* = 23)	CNS+ (*n* = 14)	*p* Value
Days from admission (days), median (IQR)	2.0 (1.0–3.0)	2.5 (2.0–5.0)	0.151
GM-CSF (pg/mL), median (IQR)	0.0 (0.0–3.4)	0.0 (0.0–25.8)	0.482
IFN-γ (pg/mL), median (IQR)	26.0 (12.2–73.4)	31.1 (19.7–51.1)	0.920
IL-6 (pg/mL), median (IQR)	11.3 (6.6–46.5)	75.8 (28.6–124.6)	0.001
IL-8 (pg/mL), median (IQR)	27.3 (12.5–43.2)	78.6 (32.1–423.0)	0.003
IP-10 (pg/mL), median (IQR)	1836 (1392–4846)	4243 (1589–7348)	0.244
MCP-1 (pg/mL), median (IQR)	181.2 (62.6–448.9)	294.2 (124.0–600.1)	0.228
RANTES (pg/mL), median (IQR)	4718 (2444–11893)	4168 (1517–9048)	0.412
VEGF (pg/mL), median (IQR)	19.9 (9.2–19.6)	21.1 (9.3–54.7)	0.835
G-CSF (pg/mL), median (IQR)	14.8 (10.8–23.0)	17.4 (14.0–39.8)	0.168
IFN-α (pg/mL), median (IQR)	30.8 (10.6–292.5)	338.7 (19.8–3779.0)	0.154
IL-10 (pg/mL), median (IQR)	15.1 (6.2–57.6)	25.7 (10.0–58.9)	0.531
IL-12p40 (pg/mL), median (IQR)	125.2 (76.4–228.5)	116.3 (63.0–169.1)	0.501
IL-13 (pg/mL), median (IQR)	2.4 (1.0–2.8)	1.9 (0.7–4.6)	0.775
IL-17A (pg/mL), median (IQR)	4.4 (3.0–10.4)	7.3 (0.5–14.5)	0.982
IL-1β (pg/mL), median (IQR)	3.6 (2.0–9.7)	3.1 (2.0–9.7)	0.871
MIP-1α (pg/mL), median (IQR)	11.7 (8.4–16.7)	14.5 (8.5–20.9)	0.531
TNFα (pg/mL), median (IQR)	2.5 (0.0–10.0)	4.2 (0.0–11.5)	0.480

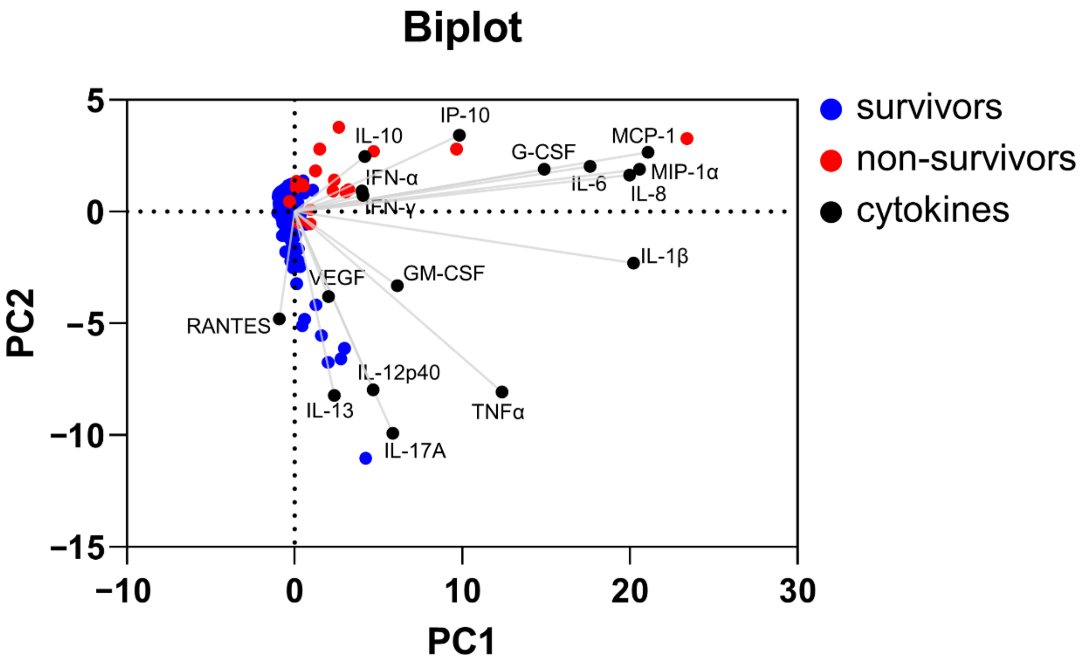

Figure S1. Principal component analysis (PCA) biplot of the entire dataset from 44 SFTS patients, plotted along PC1 and PC2.
